# Identification and quantification of viable *Lacticaseibacillus rhamnosus* in probiotics using validated PMA-qPCR method

**DOI:** 10.3389/fmicb.2024.1341884

**Published:** 2024-01-17

**Authors:** Lizheng Guo, Xiaolei Ze, Huifen Feng, Yiru Liu, Yuanyuan Ge, Xi Zhao, Chengyu Song, Yingxin Jiao, Jiaqi Liu, Shuaicheng Mu, Su Yao

**Affiliations:** ^1^China National Research Institute of Food and Fermentation Industries Co., LTD., China Center of Industrial Culture Collection, Beijing, China; ^2^Microbiome Research and Application Center, BYHEALTH Institute of Nutrition & Health, Guangzhou, China

**Keywords:** probiotics, *Lacticaseibacillus rhamnosus*, identification, viable cell quantification, PMA-qPCR method, validation

## Abstract

The identification and quantification of viable bacteria at the species/strain level in compound probiotic products is challenging now. Molecular biology methods, e.g., propidium monoazide (PMA) combination with qPCR, have gained prominence for targeted viable cell counts. This study endeavors to establish a robust PMA-qPCR method for viable *Lacticaseibacillus rhamnosus* detection and systematically validated key metrics encompassing relative trueness, accuracy, limit of quantification, linear, and range. The inclusivity and exclusivity notably underscored high specificity of the primers for *L. rhamnosus*, which allowed accurate identification of the target bacteria. Furthermore, the conditions employed for PMA treatment were fully verified by 24 different *L. rhamnosus* including type strain, commercial strains, etc., confirming its effective discrimination between live and dead bacteria. A standard curve constructed by type strain could apply to commercial strains to convert qPCR *C*_q_ values to viable cell numbers. The established PMA-qPCR method was applied to 46 samples including pure cultures, probiotics as food ingredients, and compound probiotic products. Noteworthy is the congruity observed between measured and theoretical values within a 95% confidence interval of the upper and lower limits of agreement, demonstrating the relative trueness of this method. Moreover, accurate results were obtained when viable *L. rhamnosus* ranging from 10^3^ to 10^8^ CFU/mL. The comprehensive appraisal of PMA-qPCR performances provides potential industrial applications of this new technology in quality control and supervision of probiotic products.

## 1 Introduction

Probiotics are live microorganisms which when administered in adequate amounts confer a health benefit on the host (The World Health Organization, 2001). Accurate identification and quantification of live probiotics are essential to ensure production process control and quality. *Lacticaseibacillus rhamnosus*, as one of the most popular *Lactobacillus* strains, has been widely studied because of its safety profile and desirable features of conventional probiotics (Kalliomäki et al., 2001; Mathipa-Mdakane and Thantsha, 2022; Xavier-Santos et al., 2022). *L. rhamnosus* species, e.g., LGG, HN001 etc., possess great market value in food industry attributed to their excellent fermentation performance and probiotic effect. Characteristics of tolerance to acid and bile as well as good growth ability allow them to survive and thrive within the gastrointestinal tract (De Champs et al., 2003). *L. rhamnosus* is able to form biofilms displaying as an excellent mucus-adhering *Lactobacillus* strain that enhance its ability to protect and strengthen the cytoskeleton integrity to inhibit pathogen colonization (Segers and Lebeer, 2014; Martín et al., 2019). Additionally, *L. rhamnosus* has been well documented for its clinical benefits. Many studies have reported on the use of *L. rhamnosus* GG for the prevention and treatment of gastrointestinal infections and diarrhea in children (Szajewska et al., 2007, 2011).

Compound probiotics have been applied to food, dietary supplements, infant formula, medical food, cosmetics and pharmaceuticals fields due to their generally recognized health benefits (Quin et al., 2018). Presently, lots of studies demonstrate that the efficacy of probiotics is strain-specific and disease-specific (McFarland et al., 2018). Campana et al. (2017) indicated that individual Lactic Acid Bacteria (LAB) strains showed strain-specific probiotic properties to inhibit the invasion of intestinal pathogens to Caco-2 cells. Kekkonen et al. (2008) studied a milk-based drink or a placebo drink containing *L. rhamnosus* GG (LGG), *Bifidobacterium animalis* ssp. *Lactis* Bb12 (Bb12), or *Propionibacterium freudenreichii* ssp. JS (PJS) and found that probiotics exhibited strain-specific anti-inflammatory effects in healthy adults. Additionally, the health benefits of probiotics are closely related to the amount of viable cells intake. However, viability of probiotic bacteria mostly depends on the bacterial strains, preservation methods, fermentation, and storage conditions (temperature, oxygen) (Odooli et al., 2018). Thus, it is necessary to monitor and selectively enumerate specific viable cells to ensure the stable quality of probiotic products. Currently, quantification of LAB is mainly by heterotrophic plate count methods. However, culture-based technologies are usually time-consuming (Odooli et al., 2018) and difficult to distinguish or selectively enumerate probiotics due to similar growth requirements and biochemical characteristics of multiple probiotic species in products (Ashraf and Shah, 2011). Therefore, development of species-specific detection methods for probiotic identification and enumeration are great meaningful for manufacturers to speeding up products releasing time, government product supervision and consumer rights protection.

Nucleic acid-based methods such as quantitative PCR (qPCR) have been widely applied to fields of biology, food science, environmental science for microorganisms detection as it is rapid, specific, and highly sensitive (Ceuppens et al., 2010; Portilho et al., 2018; Guo et al., 2020). However, its inability to distinguish between viable and dead cells limits its application. Fortunately, a novel dye named propidium monoazide (PMA) could be coupled with qPCR (PMA-qPCR) for viable cells quantification through selective staining based on membrane integrity (Nocker et al., 2006). The PMA dye can only penetrate membrane damaged cells and covalently cross-link with DNA during photolysis, thus preventing PCR amplification of the DNA. Consequently, DNA from membrane-intact cells could be selectively amplified by the following PCR procedure (Chiao et al., 2014; Scariot et al., 2018). The PMA-qPCR shows its advantages for selectively detecting individual strains in compound probiotic products based on species specific primer design. Several crucial factors could affect the accurate numeration of viable cells by PMA-qPCR method, such as DNA extraction method (McOrist et al., 2002; García-Cayuela et al., 2009), PMA treatment conditions (Miotto et al., 2020), construction of standard curves (Ilha et al., 2016; Odooli et al., 2018; Scariot et al., 2018), bacterial density (Zhu et al., 2012; Tantikachornkiat et al., 2016; Lu et al., 2019), etc. All these factors should be considered and confirmed its suitability to the target strains to ensure accurate results. Presently, the PMA-qPCR method has been applied to monitor viable cells of specific LAB during fermentation process or shelf life (Berezhnaya et al., 2021; Gagnon et al., 2021; Yang et al., 2021).

Microbiological methodologies necessitate the comprehensive evaluation and validation of their performance parameters, as recommended by established standards such as ISO 16140-6 (2019); United States Pharmacopeia (2023). Notably, the PMA-qPCR method offers a dual capability of enabling specific microbial identification at the genus, species, or strain level, along with the precise enumeration of viable cells. Ensuring the precision of detecting target microorganisms necessitates the rigorous validation of primer inclusivity and exclusivity. It is worth noting, however, that numerous studies frequently referenced primer sequences from existing literatures, yet often omit subsequent validation steps or inadequately encompass a comprehensive spectrum of strains, thereby leading to erroneous outcomes, such as false positives or negatives. Quantitative methodologies, including the PMA-qPCR method, demand meticulous assessment of performance parameters such as accuracy, precision, specificity, quantification limit, linearity, and ruggedness (Broeders et al., 2014). These metrics hold undeniable significance in gauging the robustness and dependability of the established methods. Although the PMA-qPCR technique has garnered widespread application across diverse sectors, encompassing fields such as food, environment, and clinical analysis, a conspicuous void remains regarding the comprehensive evaluation of its efficacy in accurately quantifying specific target species.

In this study, we developed and systematically evaluated a precise PMA-qPCR method for quantifying viable *L. rhamnosus*. Validation of the *L. rhamnosus*-specific primer included comprehensive inclusivity and exclusivity assessments through whole-genome sequence blast and strain collection at various taxonomic levels. The efficacy of PMA treatment conditions was confirmed using 24 *L. rhamnosus* strains, ensuring non-interference with viable cell PCR amplification while effectively inhibiting non-viable cells. A standard curve relating qPCR *C*_q_ values to viable bacteria numbers was established. The established PMA-qPCR method was then applied to diverse samples, revealing relative trueness, accuracy, linear, limit, quantification range. This study successfully established a robust PMA-qPCR tool for quantifying viable *L. rhamnosus* in heterogeneous samples, with implications for assessing probiotic product viability and quality.

## 2 Materials and methods

### 2.1 Inclusivity and exclusivity of primer tests

The *L. rhamnosus* specific primer sequence was Lrh-F: TGC TTG CAT CTT GAT TTA ATT TTG; Lrh-R: GGT TCT TGG ATY TAT GCG GTA TTA G (Byun et al., 2004; Mansour and Ismail, 2016). Strains of *L. rhamnosus* CICC 6224^T^, *L. rhamnosus* HN001, *L. rhamnosus* UALr-06, *Bifidobacterium animalis* subsp. *lactis* Bi-07, *B. lactis* HN019, *B. lactis* UABLa-12, *Bifidobacterium breve* M-16 V, *Bifidobacterium longum* UABL-14, *Lactobacillus acidophilus* DDS-1, *Limosilactobacillus fermentum* CECT5716, *Lactiplantibacillus plantarum* 299 V, and three products that contain *L. rhamnosus* were firstly used to validate the specificity of the primer through PCR conduction and gel electrophoresis. Positive amplification was observed on the DNA template from the three *L. rhamnosus* and the three products. No amplification occurred on the non-target strains. These results initially demonstrated the specificity of the primer to *L. rhamnosus*. Then, systemic inclusivity and exclusivity validation were performed.

Inclusivity, defined as the detection of target strains (ISO 16140-2, 2016), was firstly assessed *in silico* using the Basic Local Alignment Search Tool (BLAST®)^1^. The whole-genome sequences (WGS) of 35 *L. rhamnosus* were downloaded from NCBI website^2^. All these WGS are from bacteria including type strain, commercial strains, and others. Then, primers of *L. rhamnosus* were aligned with WGS through Primer-BLAST on NCBI^3^.

Inclusivity of primers was further tested by PCR amplification using the DNA template from 24 different *L. rhamnosus* strains (Supplementary Table S1). All the strains were firstly identified by MALDI-TOF (MBT Smart, Bruker) or 16S rRNA sequencing method to ensure the correct classification. For PCR assay (PCR system 9,700, ABI, USA), thermal cycling consisted of initial denaturation at 95°C for 5 min, followed by 35 cycles of 95°C for 30 s, 55°C for 32 s and 72°C for 25 s, followed by a final extension step of 72°C for 10 min. The amplification products were analyzed with electrophoresis on 1% agarose gel and examined under UV light (Bio-Rad Laboratories Pte. Ltd., Singapore).

Exclusivity, is defined as the non-detection of non-target strains (ISO 16140-2, 2016). Similar with inclusivity, exclusivity was also firstly assessed *in silico* using the Basic Local Alignment Search Tool. The 80 WGS of 25 *Lacticaseibacillus* on species level, 281 WGS of 30 *Lactobacillaceae* on genus level, and 72 WGS of the 35 strains in Chinese list of cultures that can be used for food were downloaded from NCBI website. Then, primers of *L. rhamnosus* were aligned with WGS through Primer-BLAST on NCBI.

Thirty-five strains in Chinese list of cultures that can be used for food were collected and further identified by MALDI-TOF or 16S rRNA sequencing method (Supplementary Table S2). The PCR amplification was conducted using the primer and the DNA templates of these strains. Then, PCR products were identified by 1% agarose gel electrophoresis.

### 2.2 Propidium monoazide treatment

Twenty-four pure culture strains of *L. rhamnosus* were chosen to verify the applicability of the PMA treatment conditions used in this study. When employing PMA, qPCR amplification of DNA from viable cells should remain largely unaffected, while DNA from dead cells should be completely inhibited. Consequently, live, and dead bacterial groups of *L. rhamnosus* were obtained for each strain, respectively. All *L. rhamnosus* strains were initially revived on MRS solid medium at 37°C for 48 h under anaerobic conditions. Subsequently, they underwent an additional 48 h of incubation after being inoculated onto MRS solid medium. Given that all *L. rhamnosus* strains were incubated twice on MRS solid medium under optimal culture conditions, most of the bacteria were presumed to be highly active. The resulting cultures were resuspended and diluted using a 0.85% sodium chloride solution. Concentrations of the resuspended bacteria were adjusted to an optical density at 620 nm (OD_620_) of 0.3–0.5, corresponding to approximately 10^8^ CFU/mL, a measure further validated by plating on MRS agar plates. Subsequently, the bacteria were categorized into live and dead groups. For the dead group, the bacteria at 10^8^ CFU/mL underwent a 20-min heat treatment at 80°C. Validation on MRS solid medium revealed no observable growth of viable cells, thus confirming the successful generation of the dead group bacteria. Both live and dead bacterial suspensions with approximate 10^8^ CFU/mL were divided into PMA treatment and non-treatment groups.

PMA (Biotium, USA) solution was dissolved in ddH_2_O to create a 20 mmol/L stock solution and 1.25 μL of that was added to 500 μL of cell suspensions to achieve final concentrations of 50 μM . The mixed samples were then placed in the dark for 5 min to allow PMA to penetrate dead cells and bind to the DNA. The treated samples were exposed to a 60 W LED light source (Biotium, USA) for 15 min. Then, both the bacterial suspensions of PMA treatment and non-treatment group were centrifuged at 12,000 *g* for 15 min. The harvested bacterial pellets were subjected to DNA extraction.

### 2.3 Genomic DNA extraction

In this study, total genomic DNA were extracted using the bead-beating (BB) method. The BEAD RUPTOR 12 (OMNI International, USA) was used as a mechanical cell disruptor. The (Zirconia/Silica) 0.1 mm beads (0.25 g) were placed in a screw-cap 2.0 mL sample tubes and both were then autoclaved. Bacterial suspension within 200 μL ddH_2_O were added into the tubes. Then, samples were lysis for 12 s at the 6.0 m/s speed setting using the bead mill homogenizer and centrifuged (15 min, 12,000 g). Fifty microliter supernatants containing DNA were taken and added into 1.5 mL sterile tubes for qPCR assay.

### 2.4 Quantitative PCR assay

The qPCR assays were performed on an ABI 7500 Fast real-time PCR system. Each 25 μL reaction mixture contained 12.5 μL of 2 × SYBR Green premix (TaKaRa, Japan), 1 μLof10μM each primer, 0.5 μL ROX, 5 μL DNA template, and 5 μL ddH_2_O. DNA samples, negative DNA control (sterile water) was included in triplicate in each qPCR run. The thermal cycle program was as follows: 95°C for 30 s, followed by 40 cycles of 95°C for 10 s, 55°C for 32 s, 72°C for 25 s.

### 2.5 Construction of PMA-qPCR standard curves

The standard curve between viable cell numbers and qPCR *C*_q_ values was made. Type strain of *L. rhamnosus* CICC 6224^T^ was initially revived on MRS solid medium at 37°C for 48 h under anaerobic conditions. Subsequently, they underwent an additional 48 h of incubation after being inoculated onto MRS solid medium. Samples were resuspended and then diluted to approximate 10^8^ CFU/mL in 0.85% sodium chloride solution. On one hand, viable cell numbers were enumerated by culture-based method. On the other hand, the 10^8^ CFU/mL bacteria solution were treated by PMA and DNA were extracted as described above. DNA solutions were diluted 10-fold in series. The diluted DNA was used to run the qPCR assay and *C*_q_ values were obtained of each dilution. Then, the standard curve between *C*_q_ values and viable cell numbers were constructed (Ilha et al., 2016; Odooli et al., 2018; Scariot et al., 2018).

### 2.6 Quantification of viable *Lacticaseibacillus rhamnosus* in a variety of samples using PMA-qPCR method

The established PMA-qPCR method was applied to detect viable *L. rhamnosus* in pure cultures, probiotics as food ingredients, and probiotic products to validate the performance of this method. The concentrations of 24 fresh cultured *L. rhamnosus*, including CICC 6224^T^, CICC 6142, CICC 20253, CICC 25096, CICC 6155, CICC 20257, CICC 20255, CICC 6143, CICC 20258, CICC 20259, CICC 21769, CICC 20061, R0011, HN001, UALr-06, MP108, GR-1, NJ551, TR08, Lr-G14, FloraActive32550, NCC 4007, FloraActive19070, LGG, were adjusted to approximate 10^8^ CFU/mL followed by PMA treatment, DNA extraction, and qPCR amplification. Meanwhile, numbers of these pure cultures were detected by the culture-based method to get the theoretical values of each strain.

The 11 probiotics as food ingredients consisted of singular *L. rhamnosus* strains (e.g., HN001, R0011, MP108, etc.) or combinations with other probiotics and lactic acid bacteria, with simple excipients like maltodextrin. The 11 compound probiotic products typically featured a more complex microbial composition, incorporating one or more *L. rhamnosus* strains in combination with one or more other probiotics and lactic acid bacteria. These compound products typically featured more intricate formulations, incorporating complex excipients such as common additives (e.g., maltodextrin, resistant dextrin, etc.), prebiotics (e.g., fructooligosaccharides, erythrosis, stachyose, etc.), and botanical ingredients (e.g., blueberries, cranberry powder, etc.). In the context of *L. rhamnosus* probiotics as food ingredients and products, a quantity of 25 g was amalgamated with 225 mL of a 0.85% sodium chloride solution to yield a bacterial suspension. Subsequently, the overall bacterial concentration was meticulously adjusted to approximately 10^8^ CFU/mL. This prepared suspension then underwent the PMA treatment protocol. Following a centrifugation step at 12,000 *g* for a duration of 15 min, the resultant pellet underwent a DNA extraction process and qPCR amplification to get the measured values of viable *L. rhamnosus*. Theoretical values of viable *L. rhamnosus* in these samples were obtained according to products claims.

Moreover, the PMA-qPCR method was applied to detect samples encompassing a wide range of concentrations, spanning from low to high levels of *L. rhamnosus*. This experimental approach involved the creation of samples by combining viable *L. rhamnosus* cells with nonviable cells of *Bifidobacterium longum* subsp. *infantis*. In each sample, a consistent count of nonviable *B. infantis* cells was maintained at approximate 10^8^ CFU/mL, while varying concentrations of viable *L. rhamnosus* cells were introduced, namely 10^8^, 10^7^, 10^6^, 10^5^, 10^4^, 10^3^, and 10^2^ CFU/mL. The quantification of viable *L. rhamnosus* cells in all samples was conducted using the established PMA-qPCR method. To assess the precision of the PMA-qPCR method, 10 replicates were performed using distinct aliquots from the same sample. Simultaneously, the culture-based method was employed to enumerate viable *L. rhamnosus* cells, obtaining the theoretical values for each sample. A comparative analysis involving linear regression was employed to examine the relationship between the theoretical and PMA-qPCR measured values, thus elucidating their linear correlation. Notably, this analysis facilitated the determination of both the quantification limit and the range of the PMA-qPCR method, further enhancing its practical applicability.

### 2.7 Statistical analysis

The *T*-test method, conducted using Excel (Microsoft Office 16), was employed to assess the significance of PMA concentrations on viable cells between the treated and non-treated groups. Additionally, the *T*-test was applied to analyze the significance between the theoretical and the measured values of all 46 samples. A significance level of *p* < 0.05 was considered to indicate a significant difference. The Bland–Altman method was applied to assess the trueness of PMA-qPCR method by R software (R version 4.2.2).

## 3 Results

### 3.1 Inclusivity and exclusivity of primer

**Figure 1:**
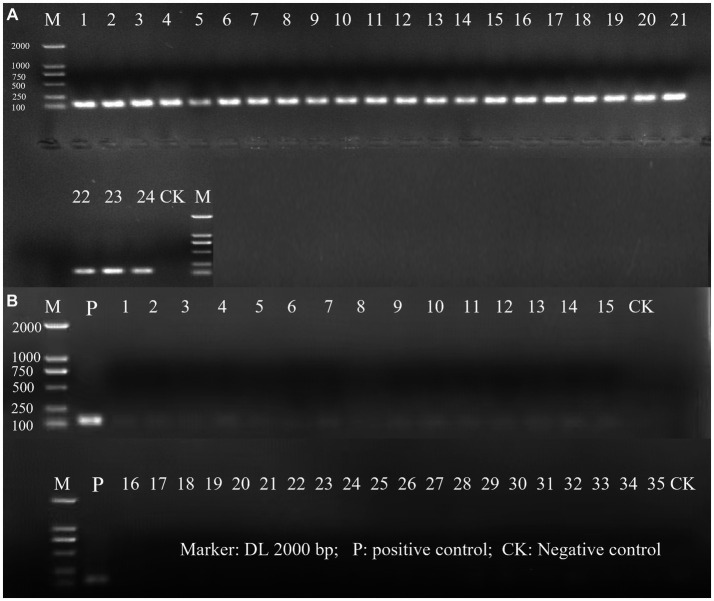
The PCR amplification of inclusivity and exclusivity assay visualized on an agarose gel. **(A)** Inclusivity assay with 24 target strains. The strain serial number is the same as in Supplementary Table S1; **(B)** Exclusivity assay with 35 non-target strains. The strain serial number is the same as in Supplementary Table S2.

The evaluation of the inclusivity and exclusivity was firstly assessed *in silico* by performing a BLAST analysis. Based on this initial test, no significant similarity with non-target microorganisms was observed. Subsequently, the specificity of the primer for detecting *L. rhamnosus* was evaluated using a PCR assay in which 24 target strains of *L. rhamnosus* and 35 non-target strains were tested. The results demonstrated that only *L. rhamnosus* strains produced a positive amplification signal, indicating that the primer was highly specific for *L. rhamnosus* and did not cross-react with other bacteria (Figure 1). The specific primer enables targeted detection of *L. rhamnosus* within multi-strain products resulting positive identification.

### 3.2 Evaluation of PMA treatment conditions on live and dead *Lacticaseibacillus rhamnosus*

The effectiveness of PMA treatment is associated with the bacterial density (Zhu et al., 2012; Tantikachornkiat et al., 2016; Lu et al., 2019), indicating that there is a specific range of bacterial density corresponding to an optimal PMA treatment conditions. In terms of experimental practicality, a total bacterial concentration of 10^8^ CFU/mL is convenient for centrifugation to acquire bacteria and facilitate subsequent DNA extraction operations. Therefore, the choice of a bacterial concentration of 10^8^ CFU/mL was made to determine the optimal PMA treatment conditions corresponding to this specific bacterial density.

**Figure 2:**
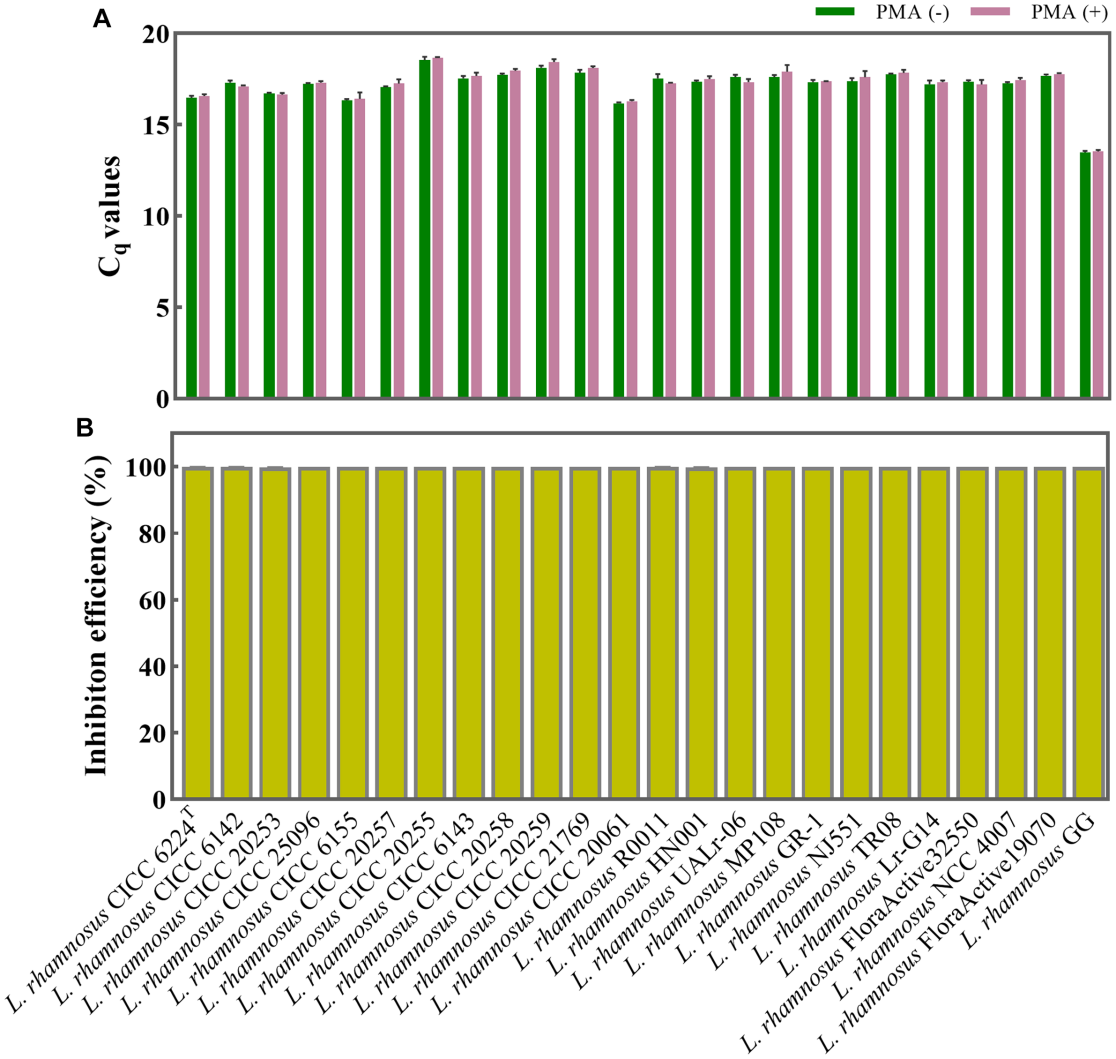
Evaluation of PMA treatment conditions for discriminating between viable and dead *L. rhamnosus* cells. **(A)** Assessment of the impact of PMA treatment on qPCR amplification of viable *L. rhamnosus* cells from 24 different strains. PMA (+) and PMA (−) represent samples treated with and without PMA, respectively. **(B)** Determination of the inhibition efficiency of PMA on dead *L. rhamnosus* cells.

To evaluate the impact of PMA treatment on viable *L. rhamnosus* cells, 24 different strains were treated with and without PMA, with each strain containing an approximate concentration of 10^8^ CFU/mL. The resulting C_q_ values in both groups were statistically analyzed using the *T*-test method. The *p* values ranged from 0.065 to 0.676 (*p* > 0.05) indicating no significant difference between the treated and non-treated groups for each strain (Figure 2A).

The PMA treatment efficiency was further evaluated when the total 10^8^ CFU/mL bacteria are all dead cells. The 24 different strains of *L. rhamnosus*, each with a concentration of 10^8^ CFU/mL, underwent heat inactivation and were subsequently divided into PMA treatment and non-treatment groups. The inhibition efficiencies of PMA treatment on qPCR amplification of dead cells from each *L. rhamnosus* strain were calculated. As shown in Figure 2B, the inhibition efficiency of each strain ranged from 99.764 to 99.994% (nearly 100%), indicating that qPCR amplification of DNA from dead cells was almost inhibited.

The PMA treatment conditions, involving a final concentration of 50 μM, a dark incubation period of 5 min, followed by light exposure for 15 min, have been identified as optimal for distinguishing between viable and dead cells of *L. rhamnosus* including the type strain, commercial strains, etc. These conditions are specifically designed to be effective under a total bacterial density of approximate 10^8^ CFU/mL. When applying this PMA treatment conditions to actual probiotic samples, it is recommended to use the optical density at 620 nm (OD_620_) method to adjust the total bacterial concentration to OD_620_ = 0.3–0.5, corresponding to an approximate concentration of 10^8^ CFU/mL.

### 3.3 Establishment of a standard curve

**Figure 3:**
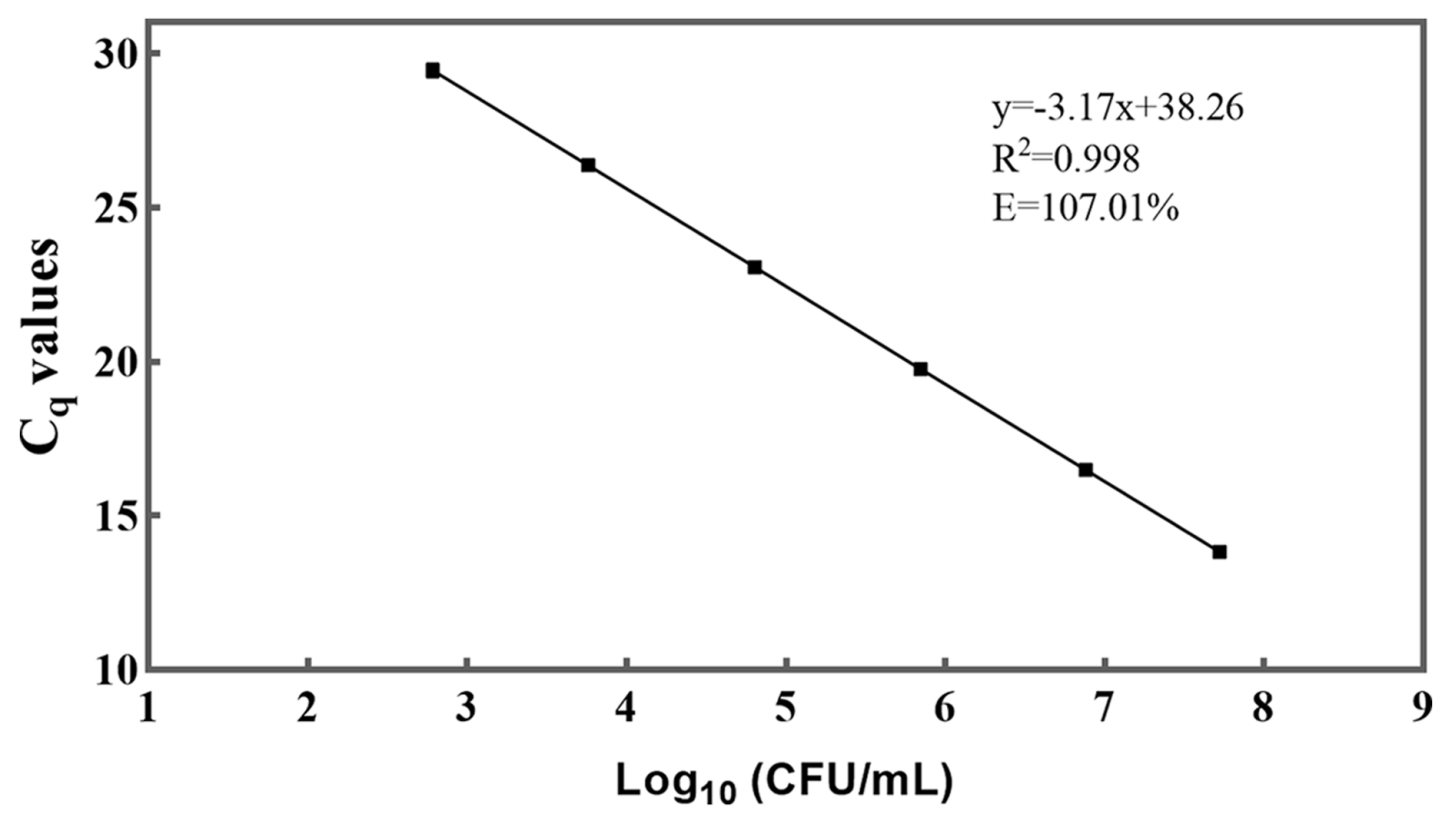
A standard curve was generated for the PMA-qPCR assay targeting *L. rhamnosus*. The plotted values on the curve represent the mean values and standard deviations obtained from three replicate tests. The C_q_ = Quantification Cycle.

A standard curve was generated by performing 10-fold serial dilutions of DNA extracted from viable *L. rhamnosus* CICC 6224^T^ cells, with culturable numbers precisely quantified at a concentration of 10^8^ CFU/mL (Figure 3) (Ilha et al., 2016; Odooli et al., 2018; Scariot et al., 2018). The generation of the standard curve involved obtaining a minimum of five concentration gradient points following the aforementioned qPCR procedure. Notably, the standard curve for DNA demonstrated a robust linear correlation (*R*^2^ = 0.998) within the approximate range of 10^3^–10^8^ genome equivalents per reaction. The high *R*^2^ value (> 0.99) indicated exceptional linearity of the qPCR assay (Elizaquível et al., 2012). Moreover, a slope of −3.17, falling within a reasonable theoretical range, was derived, and the amplification efficiency (*E*) was calculated as 107.01% using the formula *E* = 10 ^(−1/slope)^ – 1 (Fricker et al., 2007). This efficiency value is considered acceptable as it falls within the range of 90–110% (Li and Chen, 2013). These outcomes further validate the sensitivity and suitability of the primer employed for detecting *L. rhamnosus*. By utilizing the standard curve, it became feasible to convert the *C*_q_ values of *L. rhamnosus* samples into CFU equivalent cells.

### 3.4 Performance evaluation of the established PMA-qPCR method

#### 3.4.1 Applications of PMA-qPCR method to different sample types

**Figure 4:**
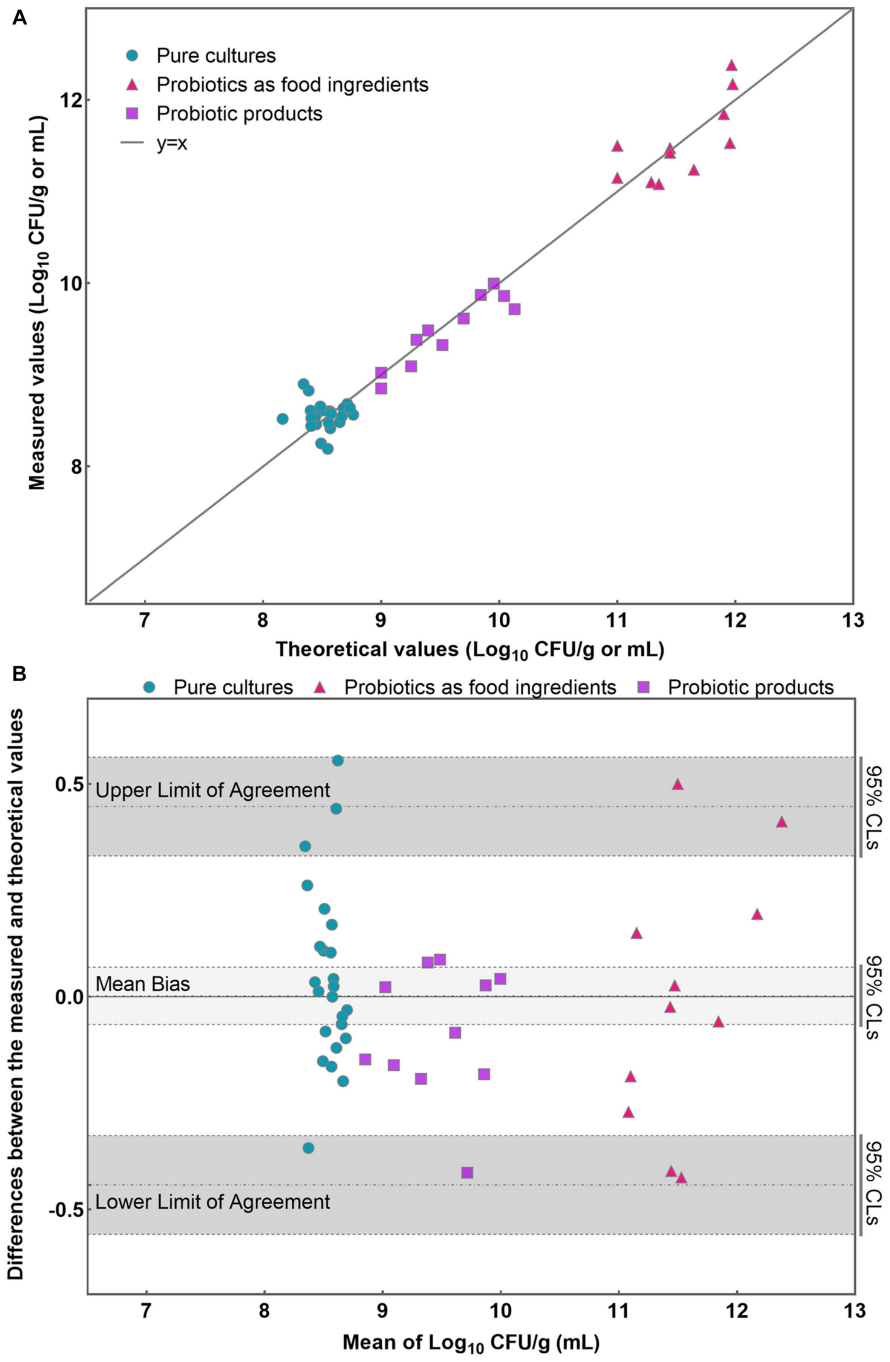
Application of PMA-qPCR method to detect viable *L. rhamnosus* in pure cultures, probiotics as food ingredients, and compound probiotic products. **(A)** Scatter plot of measured-values versus theoretical values for three different sample types; **(B)** Bland–Altman difference plot for different sample types detected by PMA-qPCR method.

Initially, a line was plotted using the data obtained from each sample, allowing for a visual assessment of the level of agreement between the theoretical and measured values (Figure 4A). Most data points closely aligned with the line for each analyzed sample, indicating a high level of concordance between the theoretical and measured values (Figure 4A). Then, the results obtained were further analyzed using the Bland–Altman method according to ISO 16140-2:2016 (E). The average of each pair of theoretical and measured values were determined and the difference (D) between the values were also calculated. Compute the average difference $\bar{D}$ for each sample, the standard deviation of differences S*_D_* and the limits of agreement using the formula $\left[\bar{D}\pm T\cdot S_{D}\sqrt{1+\frac{1}{n}}\right]$ , Where *n* is the number of data pairs, *T* is the percentile of a student-*t* distribution for β the chosen probability of the interval and (n-1) degree of freedom, that is: $T_{\left(\frac{1-\beta }{2}\right);\left(n-1\right)}$ . The individual sample differences against the mean values were plotted on a graph that shows the line of identity (zero difference), the line of bias, and the upper and lower 95% confidence limits of agreement (CLs) of the bias (Figure 4B).

The mean bias of the 46 samples was −0.003 Log_10_. The lower and upper limit of agreement were −0.442 and 0.447 Log_10_. When considering the 95% confidence limits, they were −0.558 and −0.327 Log_10_, 0.331 and 0.562 Log_10_, respectively (Figure 4B). The differences between the measured and theoretical values were −0.356 ~ 0.555, −0.425 ~ 0.500, and −0.413 ~ 0.087 Log_10_ in pure cultures, probiotics as food ingredients, and probiotic products, respectively. Evident is the remarkable coherence between the measured and theoretical values, consistently falling within the 95% confidence interval demarcated by the upper and lower limits of agreement. Furthermore, a *T*-test was employed to analyze the significance between the theoretical and measured values of all 46 samples. The resulting *p* value of 0.79 (*p* > 0.05) suggests no significant difference between the theoretical and measured groups within the 46 samples. These compellingly underscores the precision and reliability inherent in the PMA-qPCR method for the detection of viable cells across a diverse range of applications, including pure cultures, probiotics as food ingredients, and composite probiotic products.

#### 3.4.2 Applications of PMA-qPCR method to samples with different concentrations of *Lacticaseibacillus rhamnosus*

Samples with different concentrations of viable *L. rhamnosus* were prepared and detected to validate the accuracy of the established PMA-qPCR method. The accuracy profile serves as a valuable tool for assessing whether the PMA-qPCR method satisfies the criterion of generating results for a sample that deviates from theoretical values by a specific acceptability criterion. This profile facilitates the assessment of both accuracy and precision by comparing the measured values with their corresponding theoretical values. According to ISO 16140-2:2016 (E), the accuracy profile provides a comprehensive understanding of the method’s performance and its ability to meet the predefined criteria by examining the extent of agreement between these values. Typically, an acceptability limit (AL) of ±0.5 Log_10_ units is used to define the allowable difference between the measured and theoretical values (ISO 16140-2, 2016). This AL expresses the maximum acceptable deviation of the method from the theoretical values.

The data for each sample were subjected to a statistical analysis following the guidelines outlined in Step 1 to Step 9 of the Accuracy Profile study in ISO 16140-2:2016 (E). Firstly, a Log_10_ transformation was applied to the results. For each sample (i), various parameters were calculated, including the central value (Xi) representing the theoretical values, the central value (Yi) representing the PMA-qPCR results, the bias (Bi), the upper β-ETI (expected tolerance interval), and the lower β-ETI, as shown in Supplementary Table S3. The bias (Bi) was determined as the absolute difference between the medians of the theoretical and measured values (Bi = Yi – Xi). The β-ETI represents the interval within which the expected proportion of future results will fall, with β set at 80% in accordance with ISO 16140-2:2016 (E) for this study.

**Figure 5:**
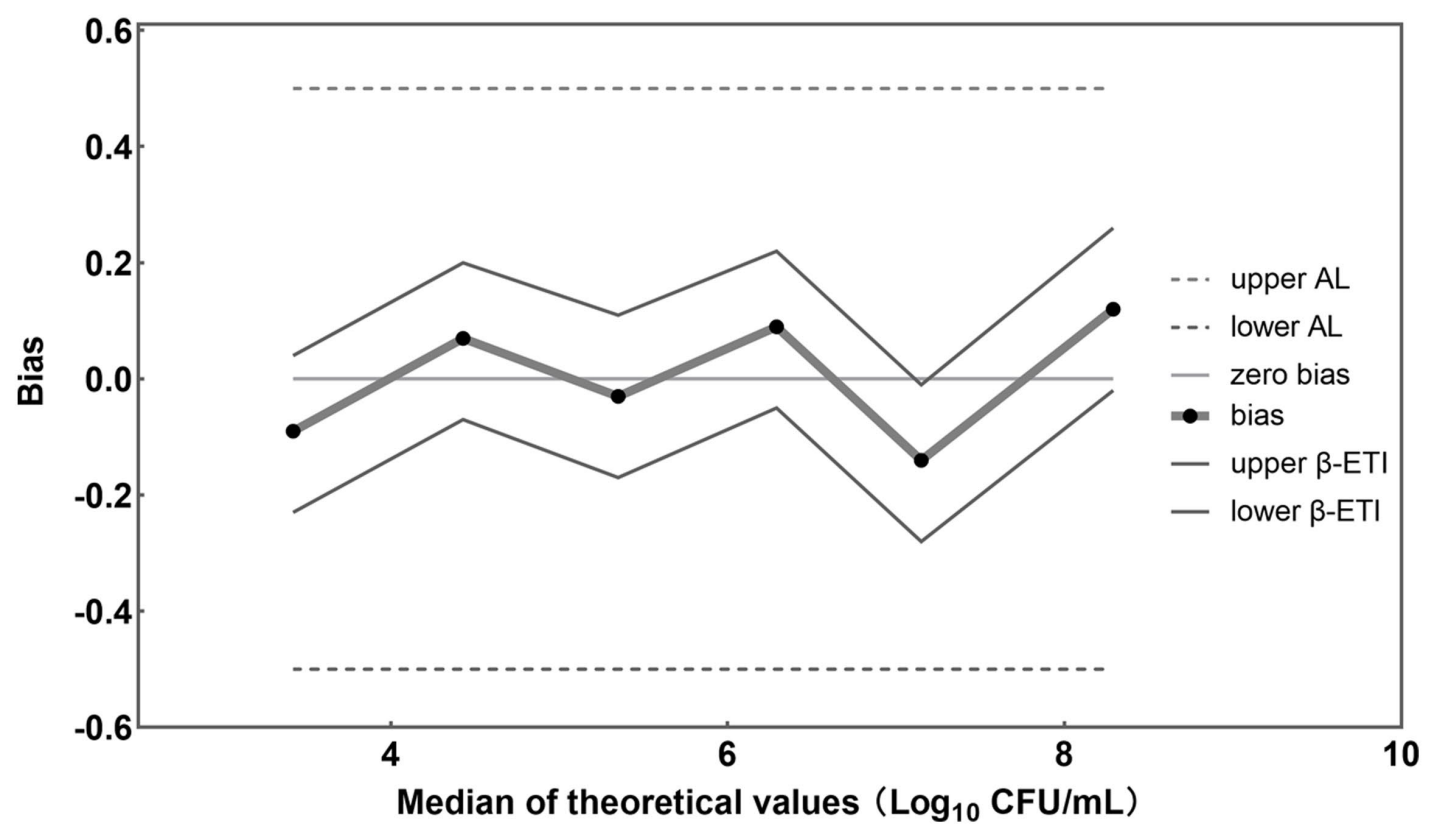
Accuracy profile for different concentrations of *L. rhamnosus* detected by the established PMA-qPCR method.

A graphical representation of computed results was made, in which the horizontal axis is for theoretical values Xi in Log_10_ units and the vertical axis is for the bias (Figure 5). The upper and lower tolerance-interval limits are connected by straight lines to interpolate the behavior of the limits between the different levels of the validation samples. The horizontal line represents the theoretical values. The differences between theoretical values and average concentration levels of *L. rhamnosus* are represented by black dots. Whenever no biases exist, these recovered values are located on the horizontal theoretical line. In addition, AL are represented by two dashed horizontal lines and β-ETI limits as broken full lines.

In this study, samples were prepared by combining *L. rhamnosus* with *B. infantis* cells to achieve a total bacterial density of approximately 10^8^ CFU/mL, with viable *L. rhamnosus* numbers ranging from 10^3^ to 10^8^ CFU/mL. The bias between theoretical and measured values for each viable cell concentration was −0.09, 0.07, −0.03, 0.08, −0.14, and 0.12 Log_10_ units (Figure 5), respectively. Importantly, all these biases were found to fall within the acceptable limits (± 0.5 Log_10_ units) (ISO 16140-2, 2016), providing evidence for the accuracy of the PMA-qPCR method in quantifying viable *L. rhamnosus* at different bacterial densities, including low, intermediate, and high levels. Furthermore, the coefficient of variation (CV) was calculated for the Log_10_-transformed viable cell counts of the 10 replicates of each sample, yielding values of 1.22, 1.75, 2.00, 0.90, 1.60, and 3.09%, respectively. These low CV values demonstrated the precision and robustness of the PMA-qPCR method in accurately quantifying viable cell counts.

#### 3.4.3 Limit of quantification, linear, and range of the established PMA-qPCR method

In this study, the limit of quantification of the PMA-qPCR method for detecting low concentrations (10^2^ and 10^3^ CFU/mL) of *L. rhamnosus* was determined. It was observed that when the *L. rhamnosus* concentration was 10^2^ CFU/mL, some of the samples exhibited *C*_q_ values higher than 30, which closely resembled the *C*_q_ values obtained from the negative controls. However, when the *L. rhamnosus* concentration was increased to 10^3^ CFU/mL, the *C*_q_ values fell within the range of the standard curve (Figure 3), indicating the validity of the data. As a result, the limit of quantification for the PMA-qPCR method was established as 10^3^ CFU/mL.

**Figure 6:**
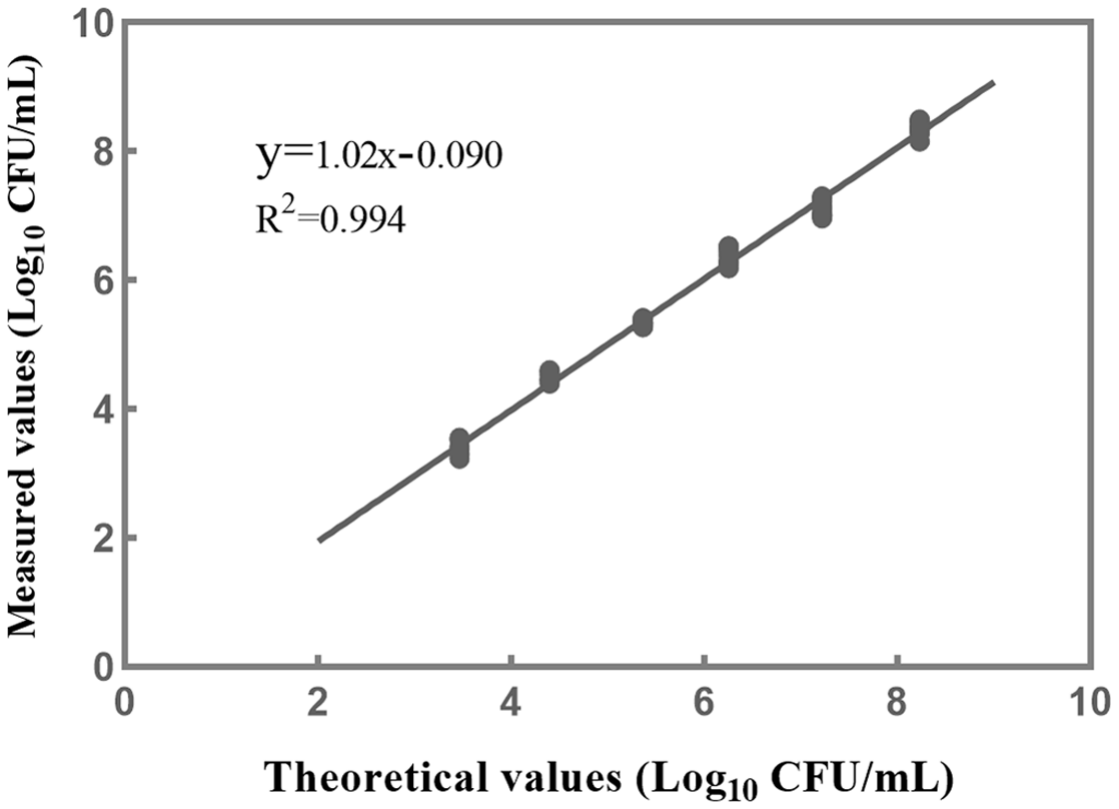
The linear fitting relationship between the theoretical and measured results when the concentrations of *L. rhamnosus* are within 10^3^–10^8^ CFU/mL. Each data point represents 10 replicates.

A linear regression analysis was performed to fit the theoretical values against the measured values (Figure 6). The resulting correlation coefficient (*R*^2^) of the fitted curve was determined to be 0.994, indicating a strong linear relationship between the measured values and the theoretical values within 10^3^–10^8^ CFU/mL (Figure 6). Based on the findings from the accuracy profile study, limit of quantification study, and assessment of linear properties, it was determined that the quantitative range for accurate detection of *L. rhamnosus* using the PMA-qPCR method is 10^3^–10^8^ CFU/mL. These results indicate that the method can provide reliable and accurate quantification within this range of bacterial concentrations. However, it is worth noting that the upper limit of the quantitative range was set at 10^8^ CFU/mL because this was the upper limit examined, and it does not necessarily represent the true upper limit of the developed method. In cases where bacterial density exceeds this concentration, the total bacterial density can be adjusted to 10^8^ CFU/mL, and the optimal PMA treatment conditions can then be applied to the samples.

## 4 Discussion

Probiotics have garnered significant attention in academic research and have found widespread usage in various food products, primarily due to their potential health benefits and their capacity to enhance gut health. Nevertheless, accurately quantifying viable cell counts in probiotic formulations containing multiple strains presents a substantial challenge (Berezhnaya et al., 2021; Yang et al., 2021). The PMA-qPCR technique has gained considerable traction for detecting viable lactic acid bacteria (Desfossés-Foucault et al., 2012; Dias et al., 2020). A critical consideration in achieving precise detection of the target microbiota through the qPCR method lies in the sensitivity and specificity of the employed primers (Broeders et al., 2014; Garrido-Maestu et al., 2018). In the current study, a comprehensive assessment of inclusivity and exclusivity effectively demonstrated the specificity of the selected primers to *L. rhamnosus* (Figure 1). Notably, these species-specific primers were meticulously designed from the V1-V2 variable regions of the 16S ribosomal DNA sequence, as denoted by its GenBank accession number AF243146 (Byun et al., 2004). This strategic design ensures the exclusivity of the primers against non-*L. rhamnosus* species (Byun et al., 2004). Furthermore, the amplification efficiency of the established standard curve was impressively high, recording a value of 107.01%. This result underscores the inherent sensitivity and reliability of the primers to accurately quantify *L. rhamnosus* (Li and Chen, 2013; Bustin and Huggett, 2017). Moreover, the substantial *R*^2^ value of 0.998 signifies a robust linear correlation between *C*_q_ values and viable bacterial counts (Figure 3). Consequently, the standard curve acquires the essential capability to translate DNA quantities into viable cell numbers (Ilha et al., 2016; Yang et al., 2021).

Effective DNA extraction is a pivotal determinant in facilitating the reliable qPCR detection of target DNA molecules within a given sample. A myriad of DNA isolation methods have been harnessed to extract DNA from bacterial source, including commercial kits (McOrist et al., 2002; Dorn-In et al., 2019), phenol-chloroform: isoamyl alcohol extraction (Vieira et al., 2021), heat treatment (Dashti et al., 2009), mechanical cell disruption (e.g., bead-beating) (Plotka et al., 2017), etc. The commercial DNA extraction kits are now mostly commonly used, which usually following by DNA purification steps. However, DNA loss during column purification has been a commonly observed phenomenon, predominantly attributed to the competitive binding of humic substances to silica membranes (Lloyd et al., 2010; Natarajan et al., 2016; Plotka et al., 2017). In the current study, DNA was extracted by only one-step lysis of cells using a bead mill homogenizer, which is rapid and easy to operate. Bead beating have been proved to effectively lysis not only Gram-negative but also Gram-positive bacteria, which have a thick cell wall (Fujimoto et al., 2004). Of paramount importance, the simplification of operational steps serves as a pivotal factor in mitigating potential DNA loss, while the consistent fixation of lysis conditions (speed and time) ensures both the stability and reproducibility of DNA quality. The effective extraction of DNA assumes primary significance as it lays the groundwork for establishing a robust correlation between *C*_q_ values and viable cell numbers, as delineated in Figure 3. High degree of consistency between theoretical and PMA-qPCR measured values of viable *L. rhamnosus* in pure cultures, probiotics as food ingredients, and compound probiotic products demonstrated the effectiveness of the DNA extraction method to different sample types (Figure 4).

Suitable PMA conditions should effectively inhibit the subsequent DNA amplification of dead bacteria without inhibiting the DNA amplification of live bacteria (Nocker et al., 2006; Fujimoto and Watanabe, 2013; Yang et al., 2021). In this study, a commonly used PMA treatment condition to LAB (Desfossés-Foucault et al., 2012; Villarreal et al., 2013) was applied to *L. rhamnosus*. One issue should be mentioned is that the effect of bacterial density on the PMA treatment efficiency should not be underestimate, as it can impact the accuracy of the test results (Zhu et al., 2012; Tantikachornkiat et al., 2016; Lu et al., 2019). Papanicolas et al. (2019) found the PMA could not fully exclude DNA amplification from dead cells with high total bacterial density, especially with high ratios of dead cells, and accurate counting of viable cells was achieved by sample dilutions (Papanicolas et al., 2019). For a defined PMA treatment condition, it applies to an appropriate cell density range (; Nkuipou-Kenfack et al., 2013; Papanicolas et al., 2019). In this study, the defined PMA treatment condition corresponds to an approximate 10^8^ CFU/mL total bacteria density, under which the PMA is enough to be very effective in modifying dead cell DNA without interfering with living cells (Figure 2). Moreover, Figure 2 confirms the wide suitability of the chosen PMA condition to commercial *L. rhamnosus* strains. These results provide reality for industrial application of the PMA-qPCR method to quantify viable *L. rhamnosus*.

The established PMA-qPCR method was used to detect viable *L. rhamnosus* in pure cultures, probiotics as food ingredients, and compound probiotic products to assess its suitability to different sample types. Figure 4 showed high consistency between theoretical and measured values of these samples, demonstrating the established PMA-qPCR method could accurately quantify viable *L. rhamnosus* in different sample types. For cells quantification by qPCR method, *C*_q_ values versus log CFU of standard curves were usually plotted using CFU by plate counting of one certain bacteria grown in culture medium (Ilha et al., 2016; Odooli et al., 2018; Scariot et al., 2018). The suitability of the standard curve made by one strain to other different strains including commercial ones within one species was not mentioned in the previous studies. The type strain CICC 6224^T^ (=ATCC 7469^T^) of *L. rhamnosus* was used to made the standard curve in this study. The commercial strains of *L. rhamnosus* in probiotics as food ingredients included HN001, R0011, MP108, UALr-06, M9, LG12-2, and in probiotic products included one or more target strains such as HN001, Lr-32, LGG, UALr-06, R0011, M9 etc. The accurate results in Figure 4 demonstrated the standard curve made by type strain could be used to quantify an unknown commercial strains of *L. rhamnosus* in probiotic samples. These results make real sense for PMA-qPCR industrial application to quantify viable *L. rhamnosus* for unknown samples or samples containing multiple *L. rhamnosus* strains. Sample matrix play an important role in the applicability of the PMA-qPCR method (Zhu et al., 2012; Miotto et al., 2020). Corresponding to probiotic products, where there are more complex matrices, e.g., excipients, prebiotics, botanical ingredients, the results showed that PMA was not significantly affected by these matrices (Figure 4). On the other hand, probiotic products contain multiple bacterial species, and PMA-qPCR can accurately target and detect viable *L. rhamnosus*, which fully demonstrates the specificity of this method. Figure 5 illustrated that the PMA-qPCR method could effectively detect viable *L. rhamnosus* cells within a range of 10^3^–10^8^ CFU/mL with high accuracy and precision, exhibiting a satisfactory linear relationship between the measured and theoretical results (Figure 6). The above results showed that the PMA-qPCR conditions established in this work can be applied to count viable *L. rhamnosus* in actual compound probiotic products, providing technical support for product quality control and supervision.

## 5 Conclusion

In this study, a PMA-qPCR method was established and validated for viable *L. rhamnosus* detection in probiotics. The inclusivity and exclusivity of the primers demonstrated its high specificity to *L. rhamnosus*, which allows accurate identification of the target bacteria. The 24 *L. rhamnosus* strains including type strain, most known commercial ones etc., confirmed the selected PMA treatment conditions could effectively distinguish between viable and dead cells. The construction of a standard curve using known quantities of type strain viable cells proved effective in converting *C*_q_ values to viable bacterial counts and it can be applied to commercial strains. The established PMA-qPCR method could quantify viable *L. rhamnosus* in pure cultures, probiotics as food ingredients, and probiotic products with high accuracy and precision. The quantitative range of the PMA-qPCR method spanned from 10^3^ to 10^8^ CFU/mL, and a strong linear relationship was observed between the theoretical and measured values within this range. The results of this study provide possible application of the PMA-qPCR method to industry for viable cell numeration of *L. rhamnosus* in compound probiotic products.

## Data availability statement

The original contributions presented in the study are included in the article/Supplementary material, further inquiries can be directed to the corresponding author/s.

## Author contributions

LG: Data curation, Methodology, Software, Validation, Visualization, Writing – original draft, Writing – review & editing. XLZ: Data curation, Funding acquisition, Methodology, Project administration, Writing – original draft, Writing – review & editing. HF: Data curation, Methodology, Validation, Writing – original draft, Writing – review & editing. YL: Data curation, Methodology, Writing – review & editing. YG: Data curation, Methodology, Writing – review & editing. XZ: Data curation, Methodology, Writing – review & editing. CS: Methodology, Writing – review & editing. YJ: Methodology, Writing – review & editing. JL: Methodology, Writing – review & editing. SM: Software, Visualization, Writing – review & editing. SY: Data curation, Funding acquisition, Methodology, Project administration, Supervision, Writing – review & editing.

## Footnotes

^1^https://blast.ncbi.nlm.nih.gov/Blast.cgi

^2^https://www.ncbi.nlm.nih.gov/genome/

^3^https://www.ncbi.nlm.nih.gov/tools/primer-blast/index.cgi?LINK_LOC=BlastHome
