# Supplementary material for: Identification and quantification of viable Lacticaseibacillus rhamnosus in probiotics using validated PMA-qPCR method
Source: Front Microbiol. 2024 Jan 17;15:1341884. doi: 10.3389/fmicb.2024.1341884 (PMC10828034; doi:10.3389/fmicb.2024.1341884)
Supplement: Supplementary file 1 [file Table_1.DOCX]

***Supplementary Material***

**Identification and quantification of viable *Lacticaseibacillus rhamnosus* in probiotics using validated PMA-qPCR method**

**Lizheng Guo^1†^, Xiaolei Ze^2†^, Huifen Feng^1^, Yiru Liu^1^, Yuanyuan Ge^1^, Xi Zhao^2^, Chengyu Song^1^, Yingxin Jiao^1^, Jiaqi Liu^1^, Shuaicheng Mu^1^, Su Yao^1*^**

^1^ China National Research Institute of Food and Fermentation Industries Co., LTD., China Center of Industrial Culture Collection, Beijing 100015, China

^2^ Microbiome Research and Application Center, BYHEALTH Institute of Nutrition & Health, Guangzhou, China

^†^These authors contributed equally to this work and share first authorship

*** Correspondence:**

Corresponding Author: Su Yao

Email: [milly@china-cicc.org](mailto:milly@china-cicc.org)

Table S1. The 24 different strains of *L. rhamnosus*.

| **Number** | **Strain** | **Identified by MALDI-TOF or 16S rRNA sequencing** | |
| --- | --- | --- | --- |
| 1 | *L. rhamnosus* CICC 6224^T^ | | *L. rhamnosus* |
| 2 | *L. rhamnosus* CICC 20053 | | *L. rhamnosus* |
| 3 | *L. rhamnosus* CICC 20061 | | *L. rhamnosus* |
| 4 | *L. rhamnosus* CICC 20255 | | *L. rhamnosus* |
| 5 | *L. rhamnosus* CICC 20257 | | *L. rhamnosus* |
| 6 | *L. rhamnosus* CICC 20258 | | *L. rhamnosus* |
| 7 | *L. rhamnosus* CICC 20259 | | *L. rhamnosus* |
| 8 | *L. rhamnosus* CICC 25096 | | *L. rhamnosus* |
| 9 | *L. rhamnosus* CICC 6155 | | *L. rhamnosus* |
| 10 | *L. rhamnosus* CICC 6142 | | *L. rhamnosus* |
| 11 | *L. rhamnosus* CICC 21769 | | *L. rhamnosus* |
| 12 | *L. rhamnosus* CICC 6143 | | *L. rhamnosus* |
| 13 | *L. rhamnosus* UALr-06 | | *L. rhamnosus* |
| 14 | *L. rhamnosus* HN001 | | *L. rhamnosus* |
| 15 | *L. rhamnosus* MP108 | | *L. rhamnosus* |
| 16 | *L. rhamnosus* R0011 | | *L. rhamnosus* |
| 17 | *L. rhamnosus* NJ551 | | *L. rhamnosus* |
| 18 | *L. rhamnosus* GR-1 | | *L. rhamnosus* |
| 19 | *L. rhamnosus* Lr-G14 | | *L. rhamnosus* |
| 20 | *L. rhamnosus* GG | | *L. rhamnosus* |
| 21 | *L. rhamnosus* TR08 | | *L. rhamnosus* |
| 22 | *L. rhamnosus* FloraActive32550 | | *L. rhamnosus* |
| 23 | *L. rhamnosus* FloraActive19070 | | *L. rhamnosus* |
| 24 | *L. rhamnosus* NCC 4007 | | *L. rhamnosus* |

Table S2. The collected 35 strains in the list of cultures that can be used for food in China.

| **Number** | **Strain** | **Identified by MALDI-TOF or 16S rRNA sequencing** |
| --- | --- | --- |
| 1 | *Lacticaseibacillus casei* CICC 6117^T^ | *Lacticaseibacillus casei* |
| 2 | *Lacticaseibacillus paracasei* CICC 6263^T^ | *Lacticaseibacillus paracasei* |
| 3 | *Lactiplantibacillus plantarum* CICC 6240^T^ | *Lactiplantibacillus plantarum* |
| 4 | *Limosilactobacillus reuteri* CICC 6132^T^ | *Limosilactobacillus reuteri* |
| 5 | *Limosilactobacillus fermentum* CICC 24209^T^ | *Limosilactobacillus fermentum* |
| 6 | *Lactobacillus delbrueckii* subsp. *bulgaricus* CICC 6103^T^ | *Lactobacillus delbrueckii* subsp. *bulgaricus* |
| 7 | *Lactobacillus acidophilus* CICC 6081^T^ | *Lactobacillus acidophilus* |
| 8 | *Lactobacillus gasseri* CICC 24878^T^ | *Lactobacillus gasseri* |
| 9 | *Lactobacillus helveticus* CICC 24208^T^ | *Lactobacillus helveticus* |
| 10 | *Lactobacillus johnsonii* CICC 6252^T^ | *Lactobacillus johnsonii* |
| 11 | *Latilactobacillus sakei* CICC 6245^T^ | *Latilactobacillus sakei* |
| 12 | *Bifidobacterium animalis* subsp. *lactis* CICC 24210^T^ | *Bifidobacterium animalis* |
| 13 | *Bifidobacterium animalis* subsp. *animalis* CICC 6250^T^ | *Bifidobacterium animalis* |
| 14 | *Bifidobacterium adolescentis* CICC 6070^T^ | *Bifidobacterium adolescentis* |
| 15 | *Bifidobacterium breve* CICC 6079^T^ | *Bifidobacterium breve* |
| 16 | *Bifidobacterium longum* subsp. *longum* CICC 6186^T^ | *Bifidobacterium longum* |
| 17 | *Bifidobacterium longum* subsp. *infantis* CICC 6069^T^ | *Bifidobacterium longum* subsp. *infantis* |
| 18 | *Bifidobacterium bifidum* CICC 6071^T^ | *Bifidobacterium bifidum* |
| 19 | *Streptococcus salivarius* subsp. *thermophilus* CICC 6222^T^ | *Streptococcus thermophilus* |
| 20 | *Lactococcus lactis* subsp. *lactis* CICC 6246^T^ | *Lactococcus lactis* |
| 21 | *Lactococcus cremoris* CICC 24337^T^ | *Lactococcus cremoris* |
| 22 | *Acidipropionibacterium acidipropionici* CICC 24923^T^ | *Acidipropionibacterium acidipropionici* |
| 23 | *Leuconostoc mesenteroides* subsp. *mesenteroides* CICC 25070^T^ | *Leuconostoc mesenteroides* subsp. *mesenteroides* |
| 24 | *Mammaliicoccus vitulinus* CICC 10850 | *Mammaliicoccus vitulinus* |
| 25 | *Lactobacillus crispatus* JCM 1185^T^ | *Lactobacillus crispatus* |
| 26 | *Staphylococcus xylosus* JCM 2418^T^ | *Staphylococcus xylosus* |
| 27 | *Latilactobacillus curvatus* JCM 1096^T^ | *Latilactobacillus curvatus* |
| 28 | *Propionibacterium freudenreichii* subsp. *shermanii* CGMCC 1.2231^T^ | *Propionibacterium freudenreichii* subsp. *shermanii* |
| 29 | *Pediococcus acidilactici* CGMCC 1.2696^T^ | *Pediococcus acidilactici* |
| 30 | *Pediococcus pentosaceus* CGMCC 1.2695^T^ | *Pediococcus pentosaceus* |
| 31 | *Weizmannia coagulans* CGMCC 1.2009^T^ | *Bacillus coagulans* |
| 32 | *Lactobacillus kefiranofaciens* subsp. *kefiranofaciens* CGMCC 1.3402^T^ | *Lactobacillus kefiranofaciens* |
| 33 | *Ligilactobacillus salivarius* CGMCC 1.1881^T^ | *Ligilactobacillus salivarius* |
| 34 | *Lactobacillus delbrueckii* subsp. *lactis* CGMCC 1.2625^T^ | *Lactobacillus delbrueckii* |
| 35 | *Staphylococcus carnosus* ACCC 01657 | *Staphylococcus carnosus* |

Table S3. Presentation of the statistical results of the comparison study (Log_10_ CFU/mL)

| **Category** | **Type** | **Concentration** | **Median of theoretical values** | **Median of PMA-qPCR measured values** | **Bias** | **Upper β-ETI** | **Lower β-ETI** | **Upper AL** | **Lower AL** |
| --- | --- | --- | --- | --- | --- | --- | --- | --- | --- |
| Samples containing  *L. rhamnosus* | Artificially formulated samples containing *L. rhamnosus* and other non-target bacteria | Low | 3.46 | 3.37 | -0.09 | 0.04 | -0.23 | 0.50 | -0.50 |
|  |  |  | 4.39 | 4.46 | 0.07 | 0.20 | -0.07 | 0.50 | -0.50 |
|  |  | Intermediate | 5.36 | 5.33 | -0.03 | 0.11 | -0.17 | 0.50 | -0.50 |
|  |  |  | 6.25 | 6.33 | 0.08 | 0.22 | -0.05 | 0.50 | -0.50 |
|  |  | High | 7.22 | 7.08 | -0.14 | -0.01 | -0.28 | 0.50 | -0.50 |
|  |  |  | 8.23 | 8.35 | 0.12 | 0.26 | -0.02 | 0.50 | -0.50 |
